# High nitrogen-containing cotton derived 3D porous carbon frameworks for high-performance supercapacitors

**DOI:** 10.1038/srep15388

**Published:** 2015-10-16

**Authors:** Li-Zhen Fan, Tian-Tian Chen, Wei-Li Song, Xiaogang Li, Shichao Zhang

**Affiliations:** 1 Key Laboratory of New Energy Materials and Technologies, Institute of Advanced Materials and Technology, University of Science and Technology Beijing, Beijing 100083, P. R. China.; 2 School of Materials Science and Engineering, Beihang University, Beijing 100191, China

## Abstract

Supercapacitors fabricated by 3D porous carbon frameworks, such as graphene- and carbon nanotube (CNT)-based aerogels, have been highly attractive due to their various advantages. However, their high cost along with insufficient yield has inhibited their large-scale applications. Here we have demonstrated a facile and easily scalable approach for large-scale preparing novel 3D nitrogen-containing porous carbon frameworks using ultralow-cost commercial cotton. Electrochemical performance suggests that the optimal nitrogen-containing cotton-derived carbon frameworks with a high nitrogen content (12.1 mol%) along with low surface area 285 m^2^ g^−1^ present high specific capacities of the 308 and 200 F g^−1^ in KOH electrolyte at current densities of 0.1 and 10 A g^−1^, respectively, with very limited capacitance loss upon 10,000 cycles in both aqueous and gel electrolytes. Moreover, the electrode exhibits the highest capacitance up to 220 F g^−1^ at 0.1 A g^−1^ and excellent flexibility (with negligible capacitance loss under different bending angles) in the polyvinyl alcohol/KOH gel electrolyte. The observed excellent performance competes well with that found in the electrodes of similar 3D frameworks formed by graphene or CNTs. Therefore, the ultralow-cost and simply strategy here demonstrates great potential for scalable producing high-performance carbon-based supercapacitors in the industry.

The depletion of fossil fuels and the increasingly environmental pollution has required the scientific community to develop new class of clean and sustainable energy sources. The fast-growing market for portable electronic devices and hybrid electronic vehicles has also demanded innovative energy storage materials of high power density and efficient energy conversion. Due to the large amount of energy stored in a very short time and long-cycle life, supercapacitors have been projected to be the most common energy storage devices^1^,^2^,^3^, with particular advancements achieved in flexible all-solid-state supercapacitors in recent years^4^,^5^,^6^,^7^,^8^.

Among the well-developed electrical double layer capacitors (EDLCs) and pseudo-capacitors, porous carbon nanostructure-based electrode materials have attracted increasing interests for supercapacitors owing to their high surface area, sufficient electrical conductivity, excellent chemical stability and low cost^9^. A remarkable variety of carbon sources with various nanostructures or unique morphologies have been widely explored, aiming to improve the electrochemical capacitance and power density. For such purpose, three-dimensional (3D) carbon porous nanostructures have been recently pursued because of the advantageous features of enhanced ion and electron transport, high specific capacities, superior electrochemical stability^10^,^11^,^12^. As typical 3D carbon nanostructures, carbon nanotube (CNT) aerogels and graphene aerogels (GAs) of high electrical conductivity, large surface area and interconnected porous structures have been largely studied^13^,^14^,^15^,^16^,^17^. For examples, Robert and coworkers have demonstrated two-electrode supercapacitor cells using 3D CNT-based materials, showing an area specific capacitance of 1 mF cm^−2^ in 1 M LiPF_6_ at a current of 10 μA^18^. On the other hand, Duan and coworkers have fabricated flexible GA-based solid-state supercapacitors, which demonstrated enhanced specific capacitance up to 186 F g^−1^ with area specific capacitance of 372 F cm^−2^ (current density of 1 A g^−1^) in two-electrode polyvinylalcohol (PVA)/H_2_SO_4_ gel system^19^. Further improvement includes simply employing heteroatoms into such 3D carbon frameworks, and thus additional pseudo-capacitance could be achieved in EDLCs. For instance, Müllen and coworkers have fabricated 3D nitrogen and boron co-doped graphene into all-solid-state supercapacitors, which showed high specific capacitance of 62 F g^−1^ at a scan rate of 5 mVs^−1^ in the two-electrode PVA/H_2_SO_4_ system^20^. Moreover, the nitrogen-doped 3D graphene framework fabricated by Qu and coworkers exhibited high specific capacitance, approaching 484 F g^−1^ at a current density of 1 A g^−1^ in 1 M LiClO_4_ aqueous solution (three-electrode system)^21^.

Although significant progresses have been made in the EDLCs based on 3D graphene- and CNT-based porous frameworks, the critical issues associated with high cost and low yield of such novel carbon-based materials still limit their applications in the industry of energy storage devices^22^. It is noticed that great challenges remain in the exploration of sufficient and simple procedures for scalable preparing CNTs and graphene^23^,^24^,^25^,^26^. As a consequence, the high price/cost of commercially available high-quality CNT and graphene-based materials (Table S1) only allows them to be used in the laboratories thus far.

In the present work, we demonstrate a simple strategy for large-scale preparing aerogel-like 3D carbon frameworks using ultralow-cost commercial cotton (0.01 USD/g, much lower than the commercially available CNTs and graphene shown in Table S1). Since the aerogel-like 3D porous framework of the cotton has been well preserved in the convenient thermal treatment, the as-prepared cotton-derived carbon frameworks (CCFs) have delivered excellent cycle stability with high specific capacities of 182 F g^−1^ at the current density of 0.1 A g^−1^ in 6 mol L^−1^ KOH aqueous solution. Further electrochemical improvements have been achieved by introducing low-cost N-doping sources (urea and melamine), and the resulting N-doped cotton-derived carbon frameworks with subsequent acid treatment (NCCFs) presented much enhanced specific capacities of 308, 240 and 200 F g^−1^ at current densities of 0.1, 1 and 10 A g^−1^, respectively. Moreover, the highest capacitance up to 220 F g^−1^ at 0.1 A g^−1^ and negligible capacitive loss were also observed in the flexible all-solid-state supercapacitors fabricated by NCCFs. Direct comparison indicates that such NCCFs of much lower cost offer very competitive electrochemical performance to the 3D graphene- and CNT-based frameworks in the literature. Implication of the results suggests that the use of extremely simple and easily scalable strategy with very cheap commercial cotton is highly promising for large-scale producing high-performance supercapacitors in the industry.

## Results

**Figure 1:**
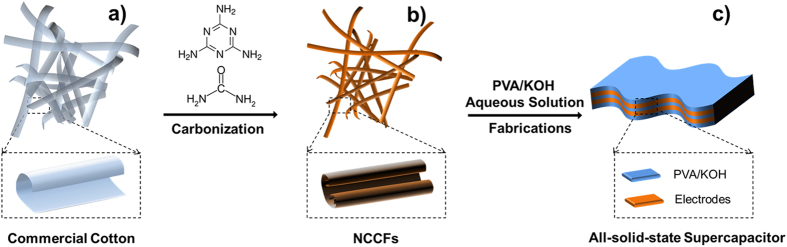
Schemes of the 3D frameworks of the as-received commercial cotton (**a**), as-prepared 3D aerogel-like carbon framework (**b**) and fabricated all-solid-state supercapacitor in the PVA/KOH gel electrolyte (**c**).

In the typical preparation (Fig. 1), certain amount of commercial cotton was used as the raw materials and the corresponding size and amount could be easily scale-up according to the chamber of furnace for carbonization. For preparing CCFs, the cotton was directly heated up for carbonization under N_2_ protection, and further treated under sonication in the mixed acid solution to improve the hydrophilicity via introducing carbonyl and hydroxyl groups^27^. NCCFs were obtained by the same conditions except for the presence of urea and melamine (N-doping sources) in the carbonization, allowing the nitrogen-containing radicals in the decomposition of melamine and urea to reacts with the carbon radicals upon high-temperature treatment.

**Figure 2:**
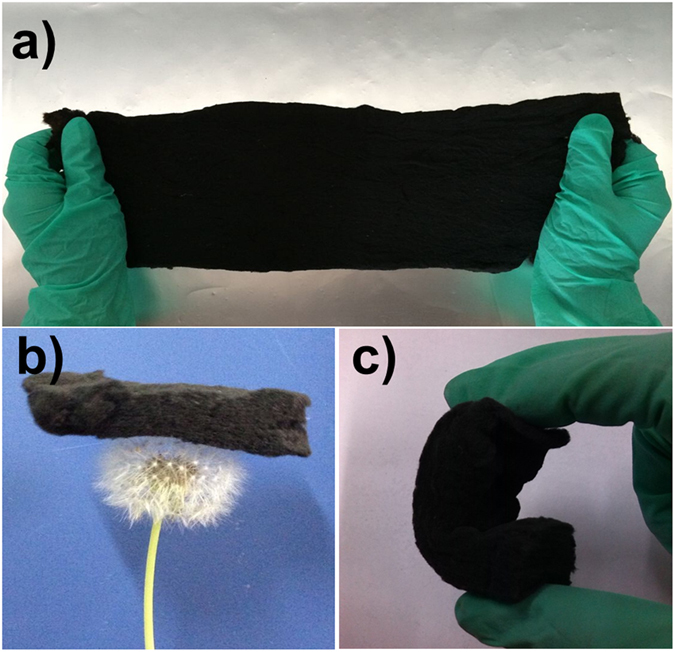
Optical images showing the scable fabrication (**a**), light weight (**b**) and mechanical flexibility (**c**) of the NCCFs.

**Figure 3:**
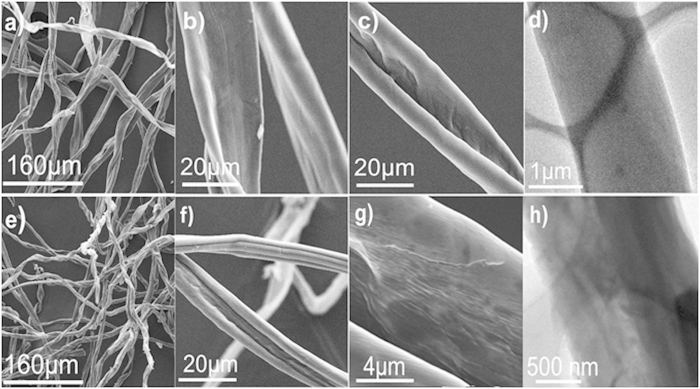
SEM images (**a–c**) and TEM image (**d**) of the commercial cotton; SEM images (**e–g**) and TEM image (**h**) of the NCCFs.

**Figure 4:**
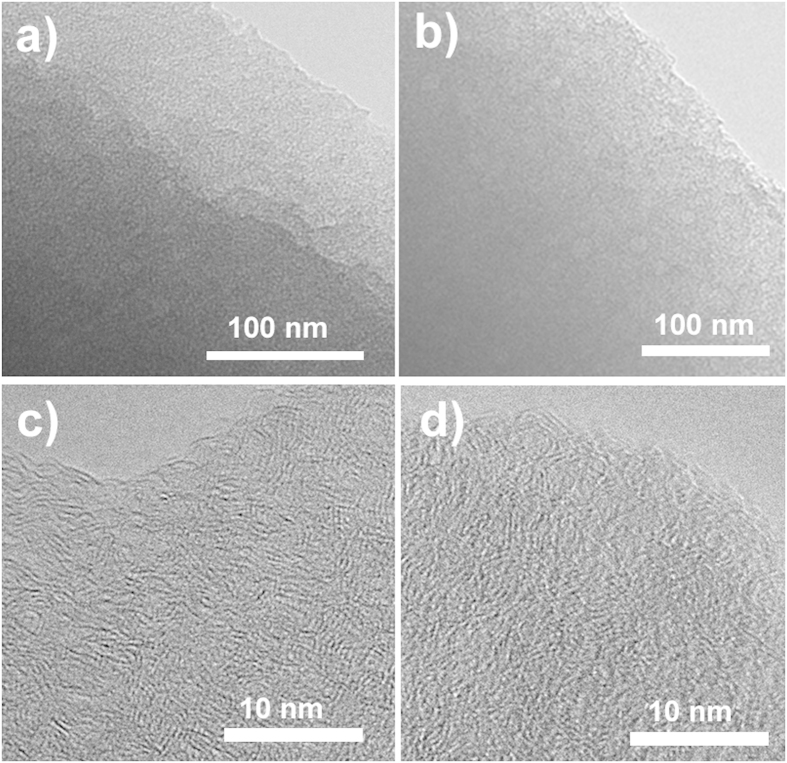
TEM images of CCFs (**a,c**) and NCCFs (**b,d**).

Figure 2a shows a large piece of NCCFs prepared in this work, indicating the easy scalability of the approach. Similar to the observation in the recently reported graphene and CNT-based 3D frameworks^28^,^29^, the as-prepared NCCFs show light weight (Fig. 2b) and mechanical robust with excellent flexibility (Fig. 2c), which has also been confirmed by the stress-strain results of the static tensile tests (Figure S1). Figure 3 exhibits typical scanning electron microscopy (SEM) and transmission electron microscopy (TEM) images of raw cotton (Fig. 3a–d) and NCCFs (Fig. 3e–h). According to the direct comparison of the morphologies, the raw cotton and NCCFs both present similar 3D frameworks, indicating that the well-formed 3D frameworks in the raw cotton has been well reserved in the high-temperature carbonization. As the basic structure for the 3D frameworks, the carbon stripes in the NCCFs exhibit much curved and twisted (Fig. 3f) in comparison with those in the raw cotton (Fig. 3b,c). Such morphological changes should be associated with the mass loss upon the high-temperature carbonization, leading to shrinkages in the curved cotton stripes as well. Representative SEM and TEM images suggest the wrinkles along with rough and porous surface of the CCFs (Figure S2) and NCCFs (Fig. 3g,h). The elemental mapping results also suggest the uniform elemental distribution of N and O in the NCCFs, due to the N-doping and acid treatments (Figure S3). According to Fig. 4, further details of the microstructures have been observed in the CCFs and NCCFs, suggesting no meaningful difference has been found in these two samples. The observed mesopores and micropores in both of the CCFs and NCCFs would provide effective porosity and improved surface area for charge storage.

**Figure 5:**
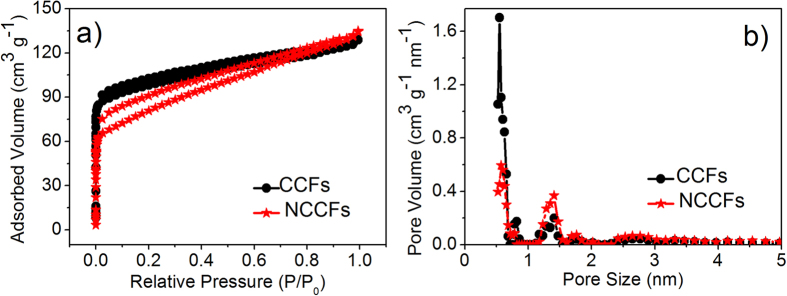
Nitrogen adsorption/desorption isortherms (**a**) and average pore diameter distributions of the CCFs and NCCFs (**b**).

Figure 5 shows the N_2_ adsorption/desorption isotherms of cotton-derived carbon materials. The pore parameters calculated from N_2_ adsorption/desorption isotherms are summarized in Table 1. According to Fig. 5a, the N_2_ adsorption/desorption isotherms of both samples exhibit type I isotherms with H4 type hysteresis loops. The rapid increase of the adsorption isotherms in the low pressure region (P/P_0_ = 0 ~ 0.1) and the hysteresis loops indicates a large amount of micropores and presence of mesopores, respectively (Fig. 5a)^30^,^31^,^32^,^33^. The results of comparison show that CCFs possess slightly larger surface area (373 m^2^ g^−1^) than NCCFs (285 m^2^ g^−1^), which is attributed to the reduced micropores caused by the micropore collapse and merge during the acid treatment. The observed larger loops suggest increased meso/macropores surface area in the NCCFs (128 cm^2^ g^−1^), compared to 88 cm^2^ g^−1^ in the CCFs. These changes are mainly attributed to the surface functionalization during the carbonization process^34^. On the other hand, the distribution of average pore diameter was obtained by the density functional theory method. As exhibited in Fig. 5b, the pores size of CCFs was mainly around 0.5 nm, while decreased peaks of 0.5 nm coupled with pronounced peaks were observed around 1.4 nm, 1.7 nm and 2.7 nm in NCCFs. Such changes indicate that the surface functionalization has not only effectively enhanced the contents of heteroatoms, but also introduced pores in the 3D frameworks as well.

**Figure 6:**
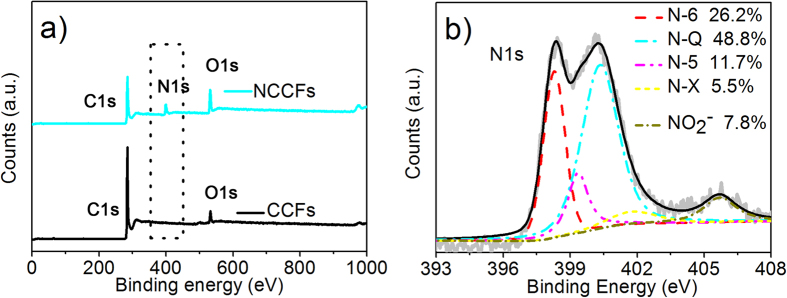
XPS spectra for CCFs and NCCFs (**a**) and N1s spectra of NCCFs (**b**).

Raman spectra (Figure S4) illustrate two prominent bands around 1360 and 1600 cm^−1^, which are denoted as the D and G bands of carbon materials, respectively^27^. No significant difference was found in the ratios of I_d_/I_g_ for CCFs (0.94) and NCCFs (0.99). X-ray photoelectron spectroscopy (XPS) was carried out to investigate the chemical composition of carbon materials (Fig. 6). As expected, pronounced C peaks have been observed in both samples (Fig. 6a), showing dominant carbon-carbon species (284.6 eV) (Figure S5). Compared to CCFs, enhanced C-N (286.33 eV) and C = O (287.9 eV) peaks have been found in NCCFs, which is attributed to the doping of nitrogen and acid treatment. It is noticeable that the substantial increase of nitrogen content in NCCFs (~12.1 mol%) listed in Table 1 suggests the sufficient N-doping with the presence of urea and melamine in the carbonization process, where nitrogen-containing precursors would react with the carbon sources to form C-N bonding under an inert atmosphere^35^,^36^,^37^,^38^. As shown in Fig. 6b, the results exhibit different types of nitrogen-containing groups in the NCCFs, and the peaks at 400.6 eV (quaternary N (N-Q) species, 48.8%) and 398.1 eV (pyridine (N-6) species, 26.2%)^38^,^39^ were generated with the presence of melamine and urea, respectively. The peak associated with nitro-type complexes NO_2_^–^ (5.5%) at 406.5 eV should be associated with acid treatment. It is well known that the positively charged N-Q and nitrogen oxide (N-X) (401.8 eV, 7.8%) could improve electron transfer, resulting in enhanced capacitive performance under larger current densities. The negatively charged pyrrole or pyridine (N-5) and N-6, on the other hand, could offer pseudo-capacitive interactions, leading to further enhancement in the specific capacitance^40^,^41^,^42^.

**Figure 7:**
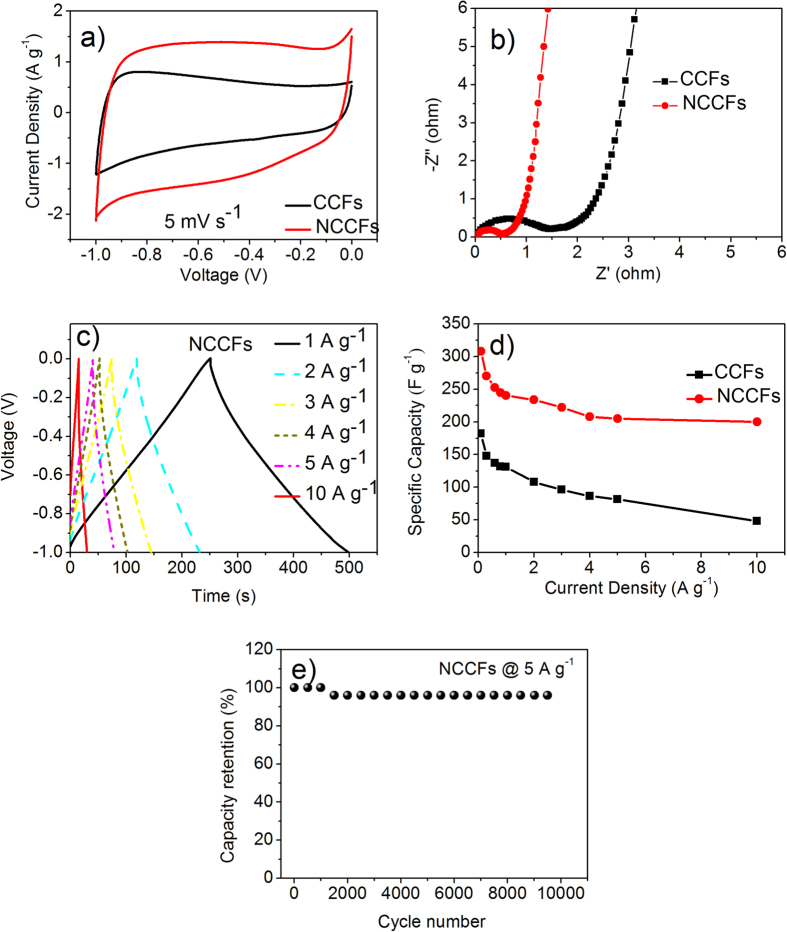
Electrochemical performance of CCFs and NCCFs measured in 6 mol L^−1^ KOH using three-electrode system: CV curves at 5 mV s^−1^ (**a**); Nyquist plots (**b**); Galvanostatic charge-discharge curves of NCCFs at different current densities (**c**); Rate performance of CCFs and NCCFs (**d**); Cycle stability of NCCFs at a current density of 5 A g^−1^ (**e**).

The results of above characterizations imply that the as-prepared NCCFs present similar morphological and structural features to the typical 3D graphene- and CNT-based frameworks. Owing to such porous 3D carbon frameworks, they should be also ideal to serve as electrode materials for supercapacitors. First, the cotton-derived 3D carbon frameworks were fabricated into a three-electrode system (6 mol L^−1^ KOH aqueous electrolyte) for electrochemical measurements. Figure 7a shows the typical cyclic voltammetry (CV) curves of CCFs and NCCFs at 5 mV s^−1^. The curves demonstrate quasi-rectangular shape with slight distortion, which is mainly induced by the pseudo-capacitance due to the oxygen and nitrogen functional groups. The redox signals of nitrogen-doped carbon electrodes are unobvious especially in alkaline electrolyte, which is in accordance with many reported works^39^,^42^,^43^,^44^. Apparently, NCCFs show much larger area than CCFs. As illustrated in Fig. 7b, the electrical resistance and ion transfer of the as-prepared supercapacitors were characterized on the electrochemical impedance spectroscopy (EIS) over a frequency range from 10^−2^ to 10^5^ Hz. According to the diameters of the semicircles at high frequency range, NCCFs exhibit lower resistance (~0.5 Ohm) than that of CCFs (~1.5 Ohm), which may be related to the promoted electron transfer with presence of N-Q and N-X. At lower frequency, all the samples present nearly vertical line, which suggests that the aerogel-like 3D porous frameworks facilitate the electrolyte transport in the cotton-derived carbon electrodes.

Figure 7c exhibits the galvanostatic charge-discharge curves of NCCFs at different current densities. The almost isosceles triangles indicate an excellent double-layer capacitance with no obvious voltage drop (IR drop) observed, which is in good agreement with the low resistance in Fig. 7b. The specific capacitance was acquired according to the equation of *C*_*S*_ = *I × t/V/m*, where C_s_ (F g^−1^) is the specific capacitance, I (A g^−1^) the response current density, t (s) the discharge time, V (V) the potential and m (g) the mass of active material. As demonstrated in Fig. 7d, the specific capacities of the NCCFs present the highest capacities, approaching 308 F g^−1^ and 200 F g^−1^ at current densities of 0.1 A g^−1^ and 10 A g^−1^, respectively. As exhibited in Fig. 7e, NCCFs shows a high retention up to 97% after 10,000 cycles at a current density of 5 A g^−1^, suggesting excellent cycle stability. It is noticed that the NCCFs with surface area only up to 285 m^2^ g^−1^ show very high capacities and excellent retention in the cycles, which should be mainly due to the high-concentration doped nitrogen (12.1 mol%) that not only introduces sufficient pseudocapacitance but also improves the electronic structure of graphitic carbon as well^45^

**Figure 8:**
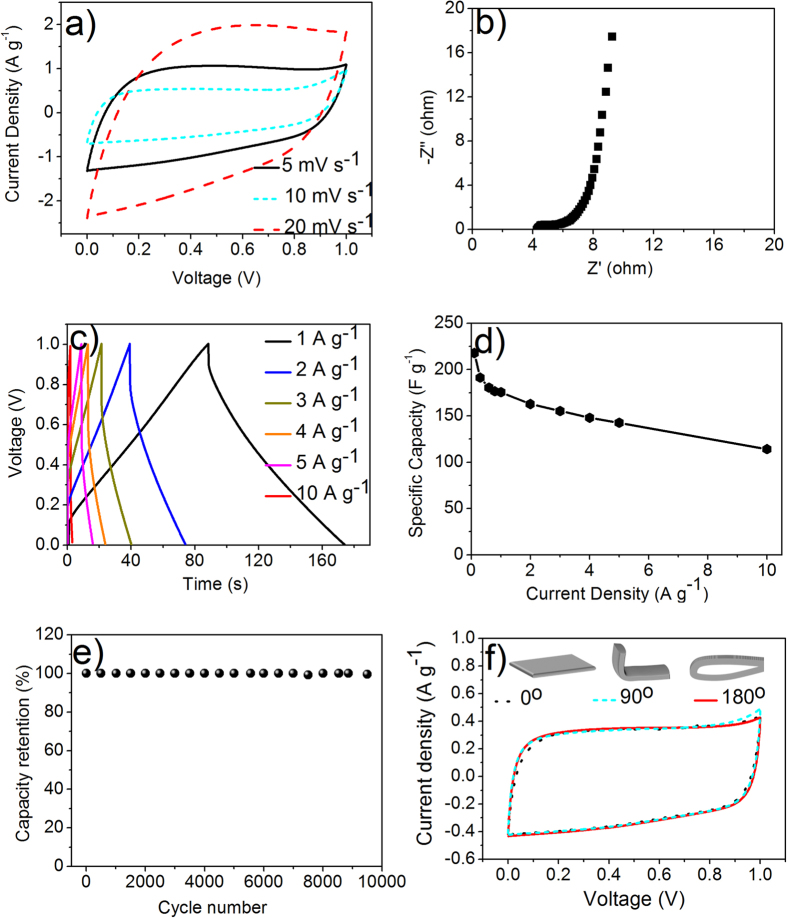
Electrochemical performance of NCCFs all-solid-state supercapacitors using PVA/KOH gel electrolyte (two-electrode system): CV curves at different scan rates (**a**); Nyquist plot (**b**); Galvanostatic charge-discharge curves at different current densities (**c**); Specific capacities at various current densities (**d**); Cycle stability at a current density of 5 A g^−1^ (**e**); CV curves at a scan rate of 5 mV s^−1^ under different bending angles (**f**).

A symmetrical flexible all-solid-state supercapacitor was further fabricated using the as-prepared electrodes coupled with PVA/KOH gel electrolyte (Fig. 1)^46^. The electrochemical properties of the all-solid-state supercapacitors are exhibited in Fig. 8. Figure 8a exhibits the CV curves at different current densities, and no pronounced redox peaks have been observed, suggesting typical double-layer capacitive behavior. The impedance in Fig. 8b shows only slight increase of impedance (~2 Ohm) in all-solid-state supercapacitors compared to the values in the three-electrode system. However, the vertical line in the low frequency region also indicates the fast ion transport in the PVA/KOH electrolyte (Fig. 8b). According to the galvanostatic charge/discharge curves in Fig. 8c, similar isosceles triangles were also observed except for the presence of limited IR drop. The specific capacitance of the electrodes in a two-electrode supercapacitor was achieved by the formula that *C*_*s*_ = 4*I × t/V/m*, where m is the mass of the active material in both electrodes. Figure 8d displays the rate stability of the all-solid-state supercapacitor, showing the highest capacitance up to 220 F g^−1^. After 10,000 cycles at a current density of 5 A g^−1^, the all-solid-state supercapacitor presented excellent cycle ability with 98% retention (Fig. 8e). In order to measure the capacitance retention under stress, the flexible supercapacitors were bent with different angles and the results show negligible effects on the capacitance upon bending (Fig. 8f).

## Discussion

The results demonstrate that the cotton-derived 3D carbon frameworks present graphene aerogel-like morphologies and structures, which allow the electrolyte to deliver fast ion transport in the porous frameworks and meanwhile enable carbon interconnected networks to serve as the effective pathways for electron transport. On the basis of the sufficient surface area of the conductive porous carbon frameworks, further enhanced capacitance could be easily achieved by the introduced N-doped functional groups. In a typical work by Ruoff and coworkers, it is suggested that the increasing N content (0.7~2.3 wt% N) would contribute to greater enhancement in the capacitive capability of the N-doped graphite oxide (Table S2)^42^. Similar results in the work by Chen and coworkers also suggest the carbon with 11.89% N content substantially enhanced the specific capacitance^43^. Therefore, such NCCFs with highly concentrated N-containing groups (12.1 wt% N) are expected to exhibit promising electrochemical performance. In the three-electrode configuration, it is interesting to find that the capacitance in NCCFs is well competitive to that found in the graphene- and CNT-based 3D frameworks (Table S2). According to the price listed in Table S1 and direct comparison of typical performance of other carbon-based electrodes, the results suggest that such low-cost 3D carbon sources with the facile strategy provide sufficient capacitance similar to the fashion carbon materials of high expense. Unlike relatively high surface areas based on the presence of CNTs and graphene in the aerogel-like structures, NCCFs here based on simply doped with high-concentration N-functional groups, which are responsible for both enhancing pseudocapacitance and facilitating electron transfer in the NCCF frameworks, have exhibited the comparable charge storage to those based on large surface areas and sufficient pore sizes.

It may be argued that the NCCFs should be directly fabricated into the binder-free electrodes for taking their advantages. In comparison, the binder-free electrode was fabricated via directly compressing the NCCF onto the Ni foam. In the same three-electrode system of 6 M KOH aqueous electrolyte, the electrochemical performance of the binder-free electrode was 173, 124 and 52 F g^−1^ at current densities of 0.1, 1 and 5 A g^−1^, respectively (Figure S6). Apparently, the binder-free systems were far behind the binder systems (308 and 200 F g^−1^ at 0.1 and 10 A g^−1^, respectively). Therefore, the binder systems can deliver more promising performance for electrochemical storage. This observation is highly usual in the biomass-based active materials^31^,^32^,^45^, which are general required to be mixed up with binders and conductive agents to fully realize the optimal electrochemical performance. Furthermore, the mass loading of the binder electrode could reach ~9 mg/cm^2^, which is more close to the practical supercapacitors. However, the mass loading of the binder-free electrode was only up to 4 mg/cm^2^ due to the highly porous framework, which may not meet the practical applications. Although the mixing process with binder and conductive agent might change the framework morphology, it is believed to be the optimized approach to fully use the capacitive capability.

Apparently, there are more opportunities for further improving the device performance because related enhancements including optimization of cotton carbonization for tuning the sp2 carbon, enlargement of the surface area and porosity or adjustment of the heteroatomic doping (N- and B-doing for examples) could be performed on the current stage, by which the fundamental understanding of the impacts on the device performance would be also achieved. Compared to the mostly attractive graphene and CNT-based 3D frameworks, the cotton-derived configurations appear to be more promising in the large-scale applications because of the much lower cost and easier scalability. Therefore, the commercially available cotton coupled with simply strategies has shown a new stage for scalable fabrication of high-performance supercapacitors.

## Conclusions

In conclusion, a facile and low-cost approach was demonstrated for scalable fabricating high-performance electrodes for supercapacitors. The commercially available cotton was used as the carbon source for preparing aerogel-like 3D nanostructures, which are favorable for both the ion and electron transport. Therefore, the resulting N-doped cotton-derived frameworks present effective capacitance, highly competitive to the performance observed in the other graphene- and CNT-based 3D frameworks. The strategy demonstrated here is very simply, low cost, easy scale-up and efficient, showing great potential for wide applications in the energy storage industry.

## Methods

### Synthesis of CCFs and NCCFs

The commercial cotton was directly used as the starting material without any further pre-treatment. Typically, a piece of cotton was cut into a certain shape (determined by the furnace size) and mixed with melamine and urea with mass ratio of 1:2:2. Subsequently, the mixture was subjected to carbonization at 800 ^o^C for 1 h with a heating rate of 5 ^o^C min^−1^ under N_2_ atmosphere. The resulting samples were further treated under ultrasonication for 1h with the presence of mixture of HNO_3_/H_2_SO_4_ (v/v = 1/3, 70% HNO_3_ and 98% H_2_SO_4_). The resulted samples (NCCFs) were then washed with distilled water and dried at 90 ^o^C overnight. For comparison, the reference samples (CCFs) were prepared under the same conditions except for the absence of melamine and urea.

Field-emission scanning electron microscopy (FESEM) was conducted on ZEISS supra 55 system. Transmission electron microscope (TEM) was performed on JEOL JEM-2010. Raman spectra were carried out on HR800 (Horiba JobinYvon) with a 514.5 nm Ar-ion laser. The nitrogen absorption/desorption isotherms associated with specific surface area and pore diameter distribution data were investigated on an Autosorb-iQ2-MP (Quantachrome) analyzer under 77k. X-ray photoelectron spectroscopy (XPS) was acquired on PHI-5300. Static tensile tests were performed with a mechanical analyzer (TA-XT Plus system, SMS).

### Characterization

The electrochemical performance of the carbon materials was determined in a three-electrode cell with basic aqueous solutions. The working electrode was prepared by mixing the carbon samples, acetylene black with polytetrafluoroethylene in a weight ratio of 80:15:5, and then the mixture was pressed onto a nickel foam. The typical mass and dimensions of the working electrodes are 10 mg and 1 cm^2^. For the three-electrode system, Pt and Hg/HgO electrode were used as the counter electrode and reference electrode, respectively, while the KOH solution (6 mol L^−1^) was employed as the electrolyte.

### Electrochemical evaluation

The electrochemical performance of NCCFs was further determined in a gel electrolyte by using a two-electrode cell. Similar to the three-electrode system, the working electrode for two-electrode system was also fabricated with the carbon samples, acetylene black and polytetrafluoroethylene (80:15:5 in weight ratio), followed by being pressed onto a nickel foam. For the two-electrode system, an all-solid-state supercapacitor was fabricated as depicted in Fig. 1c. In a typical preparation, PVA powder (2 g) was dissolved in distilled water (20 mL) under vigorous stirring at 85 ^o^C, followed by immersing two pieces of NCCFs electrodes in the PVA solution. Then, excessive KOH solution (6 M, 200 mL) was added and the mixture was kept for 24 h to form all-solid-state supercapacitors, where the PVA/KOH gel served as both electrolytes and separators.

Galvanostatic charge/discharge was tested at various current densities using LAND-CT2001A (Wuhan Jinnuo Electronics. Ltd.). Cyclic voltammetry (CV) and electrochemical impedance spectroscopy (EIS) were carried out using a CHI660C electrochemical workstation (CH Instruments, Inc.).

## Additional Information

**How to cite this article**: Fan, L.-Z. *et al.* High nitrogen-containing cotton derived 3D porous carbon frameworks for high-performance supercapacitors. *Sci. Rep.* **5**, 15388; doi: 10.1038/srep15388 (2015).

## Supplementary Material

Supporting Information

## Figures and Tables

**Table 1:** Pore parameters and chemical compositions (determined by XPS) of CCFs and NCCFs.

Samples	Pore parameters						Compositions		
	S_total_	S_micro_ (m^2^g^−1^)	S_meso_	V_total_	V_micro_ (cm^3^g^−1^)	V_meso_	C	N (mol%)	O
CCFs	373	285	88	0.20	0.11	0.09	93.8	0.7	5.6
NCCFs	285	157	128	0.21	0.07	0.14	73.4	12.1	14.6

Note: Total surface area (S_total_) and surface area of micropores (S_micro_) were obtained from multi-point Brumauer-Emmett-Teller plot and V-t plot, respectively. Surface area of meso/macropores (S_meso_) was calculated by subtracting S_micro_ from S_total_.
