# Supplementary material for: High nitrogen-containing cotton derived 3D porous carbon frameworks for high-performance supercapacitors
Source: Sci Rep. 2015 Oct 16;5:15388. doi: 10.1038/srep15388 (PMC4607951; doi:10.1038/srep15388)
Supplement: Supporting Information [file srep15388-s1.doc]

***Electronic Supplementary Information***

**High nitrogen-containing cotton derived 3D porous carbon frameworks for high-performance supercapacitors**

Li-Zhen Fan,*^a^ Tian-Tian Chen,^a^ Wei-Li Song,^a^ Xiaogang Li*^a^ and Shichao Zhang*^b^

*^a^ Institute of Advanced Materials and Technology, University of Science and Technology Beijing, Beijing 100083, P. R. China.*

*^b^ School of Materials Science and Engineering, Beihang University, Beijing 100191, China*

* Corresponding Authors: Tel/Fax: +86 10 62334311. Email: fanlizhen@ustb.edu.cn (Li-Zhen Fan); lixiaogang99@263.net (Xiaogang Li); csc@buaa.edu.cn (Shichao Zhang)

**Table S1** Price comparison of commerical cotton, graphene and carbon nanotubes.

| Products | Price  (US$/g) | Specific surface area  (m^2^/g) | Reference |
| --- | --- | --- | --- |
| SWCNTs^a)^ | 35~105 | - | [1] |
| Graphene | 98~800 | 400~1000 | [2] |
| SWCNTs | 250 | >400 | [3] |
| Graphene | 275 | >750 | [3] |
| SWCNTs | 200~2,500 | 200~400 | [4] |
| DWCNTs^b)^ | 350 | 200~400 | [4] |
| MWCNTs^c)^ | 110~150 | 200~400 | [4] |
| SWCNTs | 250 | - | [5] |
| FSWCNTs^d)^ | 2800 | - | [5] |
| FMWCNTs^e)^ | 130~370 | - | [5] |
| Graphene | 3700 | - | [5] |
| Cotton | 0.01 | >280  (carbonized) | This work |

^a)^Single-walled carbon nanotubes; ^b)^Double-walled carbon nanotubes; ^c)^ multi-walled carbon nanotubes; ^d)^ Functionalized single walled carbon nanotubes; ^e)^ Functionalized single walled carbon nanotubes.

**Table S2** Typical carbon nanomaterials and N-doped carbon materials for supercapacitors

| Electrode materials  (Type) | Specific capacitance | Electrolytes | | Refs | |
| --- | --- | --- | --- | --- | --- |
| NCCFs | 308 F g^-1^ (0.1 A g^-1^)  204 F g^-1^ (5 A g^-1^)  200 F g^-1^ (10 A g^-1^) | | 6 M KOH aqueous solution | | This work |
| mesoporous graphene nanofibers | 175 F g^-1^ (0.1 A g^-1^)  153 F g^-1^ (5 A g^-1^)  140 F g^-1^ (10 A g^-1^) | | EMIBF_4_ electrolyte | | 6 |
| CNT−CNC | 199.4 F g^-1^ (0.1 A g^-1^)  175 F g^-1^ (5 A g^-1^)  170 F g^-1^ (10 A g^-1^) | | 1 M LiPF_6_  electrolyte | | 7 |
| cMR-rGO | 195 F g^-1^ (0.1 A g^-1^)  185 F g^-1^ (5 A g^-1^)  154 F g^-1^ (10 A g^-1^) | | 1 M LiPF_6_  electrolyte | | 8 |
| N-modified FLG | 227 F g^-1^ (0.1 A g^-1^)  150 F g^-1^ (5 A g^-1^)  85 F g^-1^ (10 A g^-1^) | | 6 M NaOH electrolyte | | 9 |
| N-doped graphene | 163 F g^-1^ (0.1 A g^-1^)  152 F g^-1^ (5 A g^-1^)  148 F g^-1^ (10 A g^-1^) | | 5 M KOH aqueous solution | | 10 |
| N-doped graphene | 293 F g^-1^ (5 mV s^-1^)  236 F g^-1^ (200 mV s^-1^) | | 6 M KOH aqueous solution | | 11 |
| N-Graphene | 130 F g^-1^ (0.5 A g^-1^) | | 6 M KOH aqueous solution | | 12 |
| N-doped activated carbon | 296 F g^-1^ (2 mV s^-1^)  250 F g^-1^ (200 mV s^-1^) | | 1 M Na_2_SO_4_ aqueous solution | | 13 |
| N-doped aMEGOs-0.7% | 220 F g^-1^ (0.5 A g^-1^)  170 F g^-1^ (1 A g^-1^)  150 F g^-1^ (2 A g^-1^) | | 6 M KOH electrolyte | | 14 |
| N-doped aMEGOs-1.0 % | 255 F g^-1^ (0.5 A g^-1^)  200 F g^-1^ (1 A g^-1^)  180 F g^-1^ (2 A g^-1^) | | 6 M KOH electrolyte | | 14 |
| N-doped aMEGOs-2.3% | 350 F g^-1^ (0.5 A g^-1^)  310 F g^-1^ (1 A g^-1^)  280 F g^-1^ (2 A g^-1^) | | 6 M KOH electrolyte | | 14 |

**Figure S1** Typical stress-strain curive of the NCCFs.


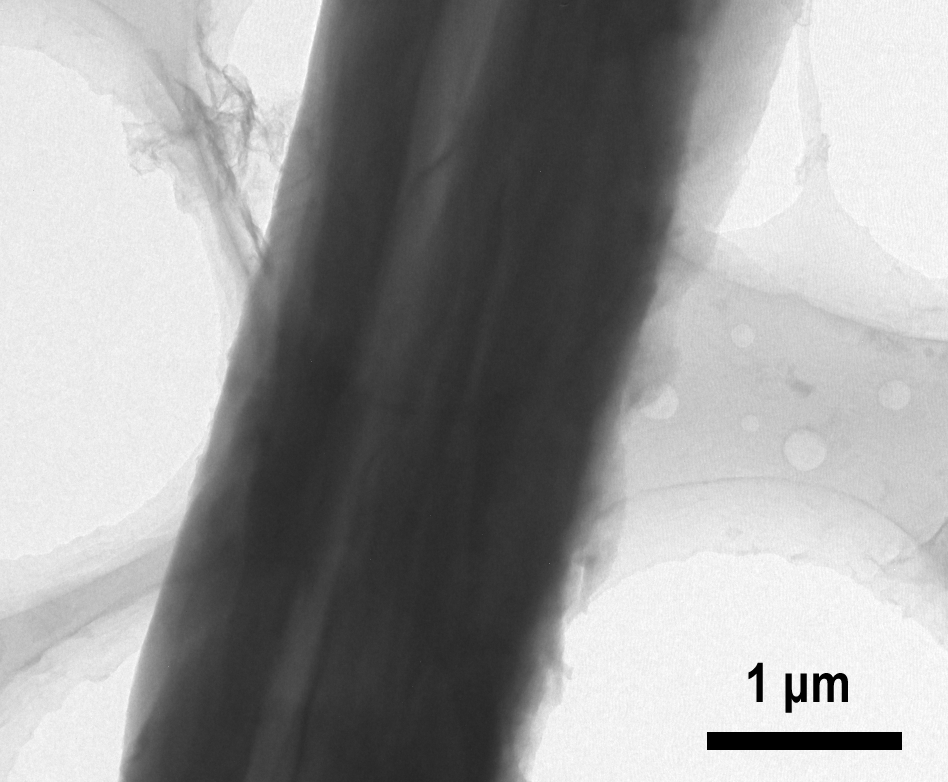


**Figure S2** TEM image of the CCFs.


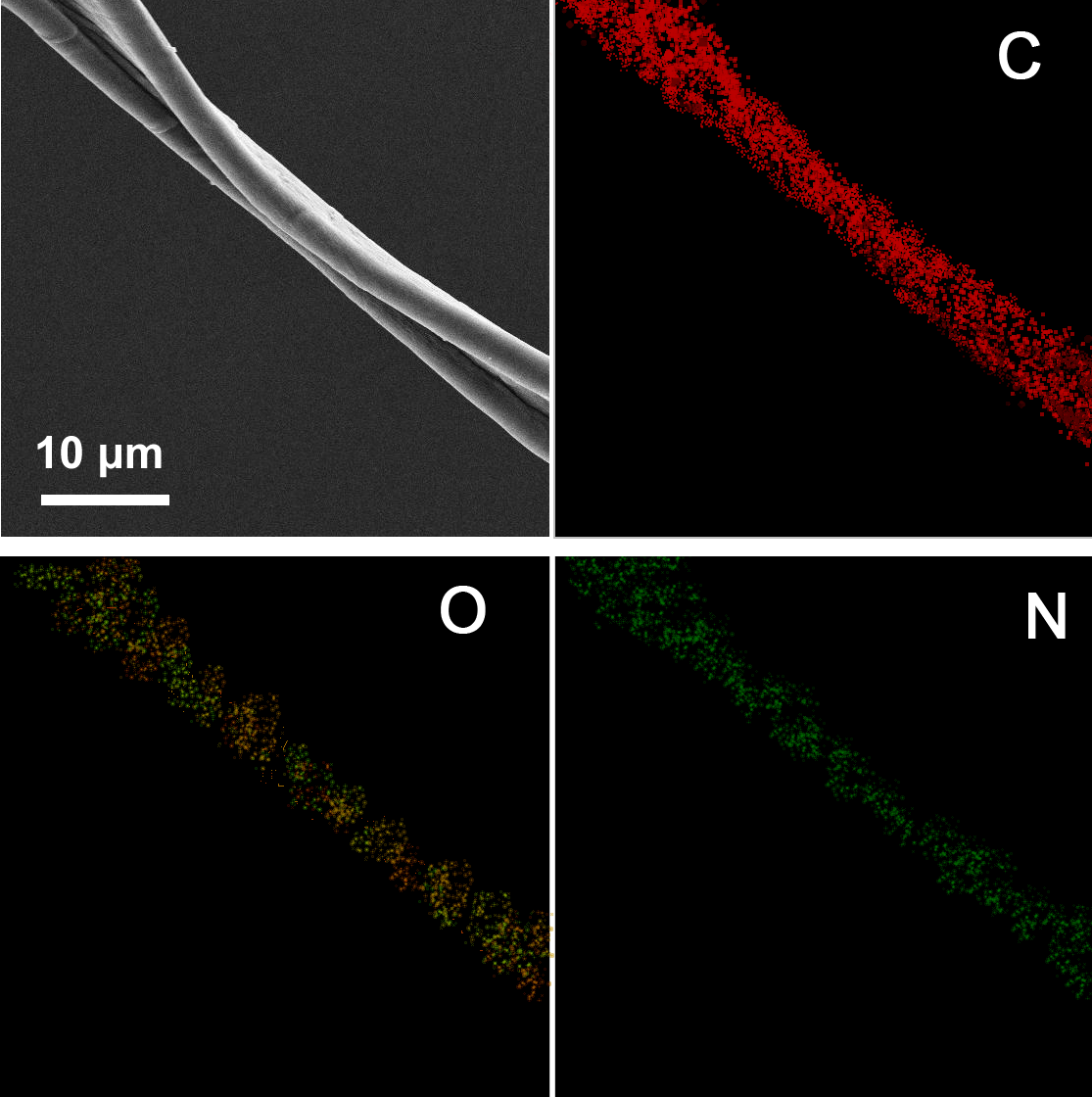


**Figure S3** Mapping of the NCCF.


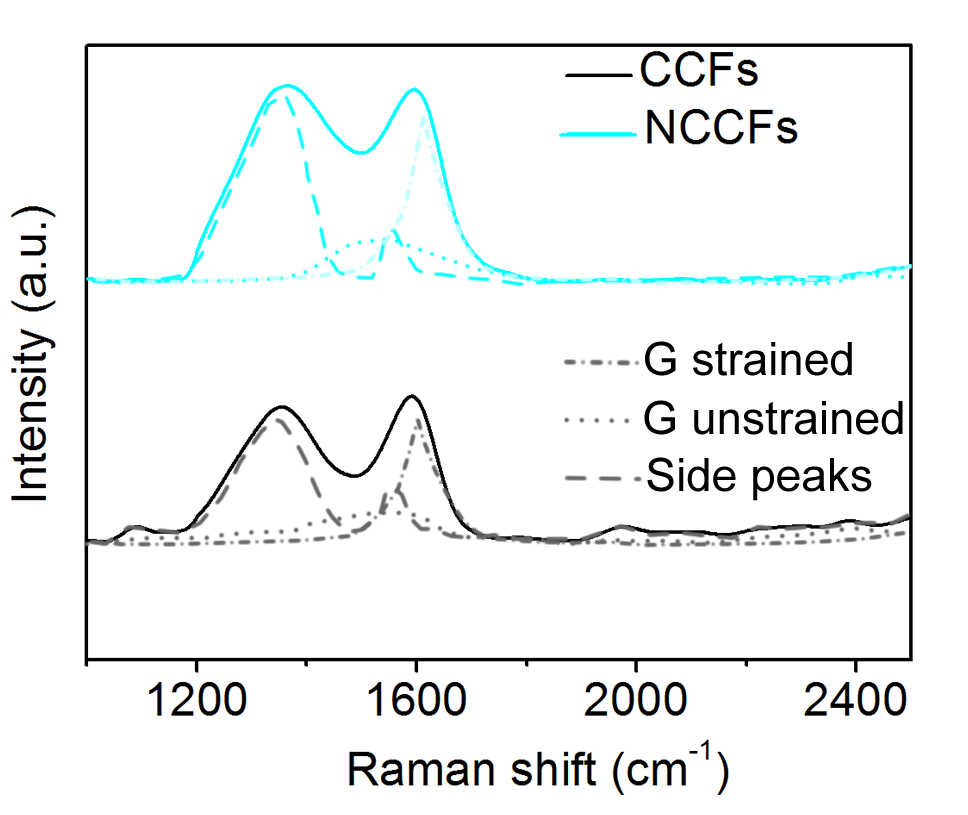


**Figure S4** Raman spectra of CCFs and NCCFs, suggesting the presence of G strained, G unstrained and side peaks (associated with D band).

**Figure S5** XPS C1s spectra of (a) CCFs, (b) NCCFs.

**Figure S6** Specific capacitance of the binder-free NCCF electrode in the three-electrode system of 6 M KOH aqueous electrolyte.

**References**

1. http://www.carbonsolution.com
2. http://www.acsmaterial.com
3. http://www.cheaptubes.com
4. http://www.nano-lab.com
5. http://www.nanocs.com/nanotube.htm
6. Cui, C.J. *et al*. Higly electroconductive mesoporous graphene nanofibers and their capacitance performance at 4V. *J. Am. Chem. Soc.* **136**, 2256-2259 (2014).
7. Hahm, M. G.  *et al*. Carbon nanotube–nanocup hybrid structures for high power supercapacitor applications. *Nano Lett.* **12**, 5616-5621 (2012).
8. Lee, J. H. *et al*. Restacking-inhibited 3D reduced graphene oxide for high performance supercapacitor electrodes. *ACS Nano* **7**, 9366-9374 (2013).
9. Xiao, N. *et al.* A simple process to prepare nitrogen-modified few-layer graphene for a supercapacitor electrode. *Carbon* **57**, 184-190 (2013)**.**
10. Cao, H. L., Zhou, X. F., Qin Z. H. & Liu, Z. P. Low-temperature preparation of nitrogen-doped graphene for supercapacitors. *Carbon* **56**, 218-223 (2013).
11. Jin, H., Wang, X. M., Gu, Z. R., Fan Q. H., Luo, B. A facile method for preparing nitrogen-doped graphene and its application in supercapacitors. *J. Power Sources* **273,** 1156-1162 (2015).
12. Lee, K. H. Oh, J. W., Son, J. G., Kim, H., Lee, S. S., Nitrogen-Doped Graphene Nanosheets from Bulk Graphite using Microwave Irradiation. *ACS Appl. Mater. Interfaces* **6**, 6361-6368 (2014).
13. Long, C. L., Qi, D. P., Wei, T., Yan, J., Jiang, L. L., Fan, Z. J., Nitrogen-Doped Carbon Networks for High Energy Density Supercapacitors Derived from Polyaniline Coated Bacterial Cellulose. *Adv. Funct. Mater.* **24,** 3953-3961 (2014).
14. Zhang, L. L. *et al.* Nitrogen doping of graphene and its effect on quantum capacitance, and a new insight on the enhanced capacitance of N-doped carbon. *Energy Environ. Sci.* **5**, 9618-9625 (2012).
